# Relationship between bone mineral density and fragility fracture risk: a case-control study in Changsha, China

**DOI:** 10.1186/s12891-021-04616-8

**Published:** 2021-08-24

**Authors:** Hong-Li Li, Yi Shen, Li-Hua Tan, Song-bo Fu, Ru-Chun Dai, Ling-Qing Yuan, Zhi-Feng Sheng, Zhong-Jian Xie, Xian-Ping Wu, Er-Yuan Liao, Xu-Lei Tang, Xi-Yu Wu

**Affiliations:** 1 National Clinical Research Center for Metabolic Diseases, Department of Metabolism and Endocrinology, Hunan Provincial Key Laboratory for Metabolic Bone Diseases, The Second Xiangya Hospital, Central South University, No.139 Middle Renmin Road, 410011 Changsha, Hunan PR China; 2 Department of Endocrinology, The First Hospital of Lanzhou University, No.1 DongGang West Road, 730000 Lanzhou, Gansu Province PR China; 3 Department of Orthopaedics, The Second Xiangya Hospital, Central South University, No.139 Middle Renmin Road, 410011 Changsha, Hunan PR China; 4 Department of Radiology, The Second Xiangya Hospital, Central South University, No.139 Middle Renmin Road, 410011 Changsha, Hunan PR China

**Keywords:** Fragility fracture, Osteoporosis, Bone mineral density, *T*-score, Fracture risk

## Abstract

**Background:**

Fragility fracture is associated with bone mineral density (BMD), and most databases used in related researches are instrument-matched. Little is known about the relationship between BMD and fragility fracture risk of native Chinese, especially using local databases as reference databases.

**Objective:**

To investigate relationship between BMD and risk of fragility fracture in native China.

**Methods:**

3,324 cases, including 2,423 women (67.7 ± 8.9 years) and 901 men (68.4 ± 11.6 years) having radiological fragility fractures and 3,324 age- and gender-matched controls participated in the study. We measured BMD at posteroanterior spine and hip using dual-energy X-ray absorptiometry (DXA), calculated BMD measurement parameters based on our own BMD reference database.

**Results:**

BMDs and mean *T*-scores were lower in case group (with clinical fragility) than in control group (without clinical fragility). In patients with fragility fractures, prevalence of lumbar osteoporosis, low bone mass, and normal BMD were 78.9 %, 19.3 %, and 1.8 %, respectively, in women, and 49.5, 44.8 %, and 5.7 %, respectively, in men. In hip, these prevalence rates were 67.2 %, 28.4 %, and 4.4 % in females, and 43.2 %, 45.9 %, and 10.9 % in males, respectively, showing differences between females and males. Multivariate Cox regression analysis showed that after adjusting age, height, weight, and body mass index, fracture hazard ratio (HR) increased by 2.7–2.8 times (95 % CI 2.5–3.1) and 3.6–4.1 times (95 %CI 3.0–5.1) for women and men respectively with decreasing BMD parameters. In both sexes, risk of fragility fracture increased approximately 1.6–1.7 times (95 % CI 1.5–1.8) for every 1 *T*-score reduction in BMD.

**Conclusions:**

Risk of clinical fragility fracture increases with decreasing BMD measurement parameters and anthropometric indicators in native China, and fracture HR varies from gender and site.

## Background

Osteoporosis is an age-related disease, characterized by decreased bone mass and damage to the microstructure of bone tissue, leading to decreased bone strength and increased risk of fractures. The main serious consequence of osteoporosis is fragility fracture. Fifty-six million people worldwide were estimated to have sustained a fragility fracture in 2000 [1]. Fractures may cause disability and loss of self-care ability, and even result in death [2–5]. Severe fractures are estimated to cause up to 30 % additional mortality [4]. Osteoporosis and fragility fractures entail a heavy economic burden on patient’s family and society as a whole [6–9]. Epidemiological surveys have shown that bone mineral density (BMD), prevalence of osteoporosis, and incidence of fragility fractures, differs among different racial and ethnic groups and regions [10–15]. For example, the prevalence of osteoporosis and incidence of fragility fractures are much higher in white people than in black people [10, 11]. Canadians have a significantly lower incidence of hip fracture than Americans and Germans [13]. The incidence of fragility fractures is lower in Asians than in white people [10, 12], while that of fragility fractures is much lower in Beijing than in the rest of China, Taiwan and Hong Kong [14, 15].

In developed western countries such as the United States and Canada, incidence of fragility fractures in white people is declining year by year [3, 16, 17]. However, in developing countries such as China, incidence of fragility fractures is continuously and rapidly increasing with escalating urbanization and rapid aging of the population [15, 18]. It is estimated that by 2050, half of fragility fractures worldwide will occur in Asia, mainly in China [19]. Prospective studies have shown that low BMD is the main important determinant of fragility fractures [20, 21], and dual-energy X-ray absorptiometry (DXA) measurements of BMD are a powerful predictor of the risk of fragility fractures in individuals of different races [22, 23], because this risk increases with decreasing BMD. Many studies have shown that fragility fracture risk will increase by one-to-three times if BMD reduced by one standard deviation [24–26]. However, in mainland China, the relationship between BMD and fragility fracture risk in vulnerable bone sites, such as lumbar spine and hip, remains unknown. Most of the databases used in other related articles are instrument-matched reference databases, derived from different ethnicities and regions, these factors will affect validity of BMD [27, 28]. In our laboratory, we have established a proprietary BMD reference database, which is more reliable for detection for southern Chinese compared with instrument-matched Asian reference databases [27, 28]. We conducted this research on our database and obtained the authentic BMD *T*-scores. We used DXA to measure BMDs and *T*-scores of lumbar spine and hip in 3,324 patients with a variety of clinical fragility fractures as well as the 3,324 controls. Based on these data, the purpose of this paper is to understand relationship between BMD measurement parameters and risk of fragility fractures in mainland China, as well as the distribution of BMD *T*-scores and prevalence of osteoporosis in fragility fracture patients.

## Methods and materials

### Study design and population

The study was performed in Xiangya Second Hospital, Central South University, Changsha, China, from March 2011 to December 2018. Patients with a variety of fractures diagnosed on radiography in our hospital were potential subjects for case group. The inclusion criteria for patients with fragility fractures were symptomatic vertebral or other fractures sustained with little damage (i.e., falls from standing or less than standing height) or without falling down. Vertebral fractures were confirmed on lateral vertebral radiographs in accordance with semi-quantitative method [29]. Patients with following conditions were excluded: traumatic fractures caused in accidents (such as car accidents or falls from above chair height), pathological fractures due to cancer, and fractures of fingers, toes, ankles, face or skull. A total of 3,324 patients, including 2,423 females and 901 males, met the inclusion criteria were included. Of these, 2,577 were spinal fractures, 360 were femoral neck fractures, and 387 were other fractures (such as forearm, wrist, proximal humerus, rib, tibia, and shoulder fractures, but excluding spine and hip fractures).

We also recruited 3,324 community residents as control group from Changsha City. The control group consisted of people who had received physical examination or sought medical care in our hospital. The control and case groups were matched for age and gender in 1:1 ratio. The inclusion criteria for control group were as follows: no history of low- or high-damage fractures and no vertebral deformities on lateral vertebral radiographs. Subjects with osteosclerosis and abnormal increase in BMD and those with skeletal fluorosis were also excluded. This study was approved by the Ethics Committee of Second Xiangya Hospital affiliated to Central South University, and each participant signed an informed consent.

### BMD measurement

BMDs and *T*-scores were measured with DXA fan-beam bone densitometer (Hologic Delphi A; Hologic, Bedford, MA, USA) at posteroanterior (PA) spine (L1–L4) and hip. If vertebral body of the fracture patients was artificial bone cement filler or metal supports after surgery, these vertebrae were excluded in analysis. If patient has a fracture or artificial joint replacement surgery on left femoral neck, measured the right hip. If bilateral femoral neck fractures on the patient, abandon the hip measurement. Using the Root mean square coefficient of variation (RMSCV) method, the in vivo accuracy deviations measured by twice BMD in 33 subjects were 0.86 % for PA spine and 0.83 % for total hip. The long-term (> 17 years) coefficient of variation for daily controlled spinal phantom scans was less than 0.45 %. Sex-specific BMD *T*-scores were calculated using our own female and male BMD reference database [27, 28]. According to the World Health Organization (WHO) definition [30], compared with peak BMD of the same gender, if subjects BMD *T*-score > − 1.0, diagnosed as normal BMD; *T*-score ≤ − 1.0 to > − 2.5 and ≤ − 2.5, were determined to be osteopenia and osteoporosis, respectively.

### Statistical analysis

All calculations were performed using SPSS V17.0 for Windows software (SPSS Inc., Chicago, IL, USA). The one-way ANOVA was used to compare the differences of BMD and *T*-scores between case groups (vertebral fracture, hip fracture, and other fractures groups) and control groups. Cox proportional hazard regression models were used to analysis association between different factors and fragility fractures risk. We used univariate and multivariate analysis to calculate hazard ratio (HR) with their corresponding 95 % confidence interval (CI). In multivariate analysis, we adjusted for age, age at menopause, years since menopause, body height, body weight, and body mass index. χ^2^ test was used to compare the prevalence of osteoporosis, osteopenia, and normal BMD between men and women. *P* < 0.05 was taken to indicate a statistically significant effect.

## Results

### Baseline characteristics

Baseline characteristics of the study subjects are shown in Table 1. Since the age of each individual in case group was exactly matched with that of control subject, the average ages of women and men in case group were identical to those in control group. The average ages of women and men in case group were 67.7 ± 8.9 years (range, 45–95 years) and 68.4 ± 11.6 years (range, 45–93 years) respectively. For both men and women, BMD at different skeletal sites and mean *T*- scores were significantly lower in case group than in control group. The mean lumbar spine BMD in both women and men were significantly lower in vertebral fracture subgroup than in hip fracture and other subgroups. The average BMD and *T*-scores at different skeletal sites were significantly greater in other fracture subgroup than in vertebral and hip fracture subgroups.

**Table. 1:** Comparison of baseline characteristics among cases of fractures and controls

Variable	Control	Case	Fracture subgroup		
			Vertebral fracture	Hip fracture	Other fractures
**Female**					
* n*	2423	2423	1819	268	336
Age (years)	67.7 ± 8.9	67.7 ± 8.9	68.2 ± 8.8	69.4 ± 9.0	63.7 ± 8.3^c^
PA-BMD (g/cm^2^)	0.822 ± 0.132	0.629 ± 0.111^a^	0.609 ± 0.102^b^	0.660 ± 0.109	0.712 ± 0.113^c^
Hip-BMD (g/cm^2^)	0.743 ± 0.115	0.584 ± 0.113^a^	0.574 ± 0.110	0.566 ± 0.100	0.650 ± 0.113^c^
PA *T*-score	–1.48 ± 1.19	–3.24 ± 1.00^a^	–3.42 ± 0.92	–3.26 ± 0.85	–2.49 ± 1.02^c^
Hip *T*-score	–1.38 ± 1.12	–2.93 ± 1.10^a^	–3.02 ± 1.08	–3.10 ± 0.98	–2.28 ± 1.10^c^
**Male**					
* n*	901	901	758	92	51
Age (years)	68.4 ± 11.6	68.4 ± 11.6	68.4 ± 11.5	70.5 ± 12.9	65.0 ± 10.2^c^
PA-BMD (g/cm^2^)	0.914 ± 0.114	0.718 ± 0.113^a^	0.707 ± 0.103^b^	0.761 ± 0.148	0.803 ± 0.127^c^
Hip-BMD (g/cm^2^)	0.865 ± 0.101	0.693 ± 0.123^a^	0.689 ± 0.117	0.661 ± 0.141	0.807 ± 0.111^c^
PA *T*-score	0.05 ± 1.02	–2.51 ± 1.01^a^	–2.61 ± 0.92^b^	–2.12 ± 1.32	–1.75 ± 1.14^c^
Hip *T*-score	–0.09 ± 0.92	–2.31 ± 1.12^a^	–2.34 ± 1.07	–2.60 ± 1.29	–1.27 ± 1.01^c^

*PA-BMD* posteroanterior (PA) spine bone mineral density

^a^*P* = 0.000 compared with control

^b^*P* = 0.005–0.000 compared with hip and other fractures

^c^*P* = 0.005–0.000 compared with vertebral and hip fractures

### Relation of BMD ***T***-score with age

**Fig. 1:**
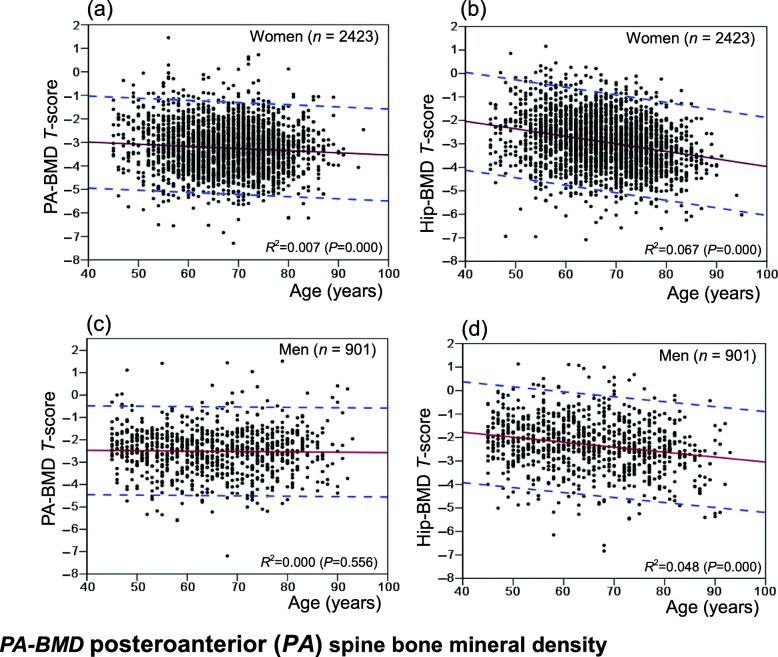
Distributions of *T*-scores and 95 % CI of age-related changes in patients with osteoporotic fractures

Figure 1 shows the distribution and 95 % confidence interval of BMD *T*-scores of fracture patients with age, of which female fracture spine and hip *T*-scores decline with increasing age. In 95 % of patients with various fragility fracture, lumbar spine *T*-scores and hip *T*-scores range from − 0.74 to − 5.74 and − 0.18 to − 5.68. In male fracture patients, lumbar spine BMD *T*-scores do not change with age, while hip BMD *T*-scores decrease with increasing age. 95 % of fracture patients have lumbar spine BMD *T*-scores between 0.01 and − 5.04, and hip BMD *T*-scores between 0.49 and − 5.11.

### Rates of osteoporosis, osteopenia, and normal BMD in fractures

**Fig. 2:**
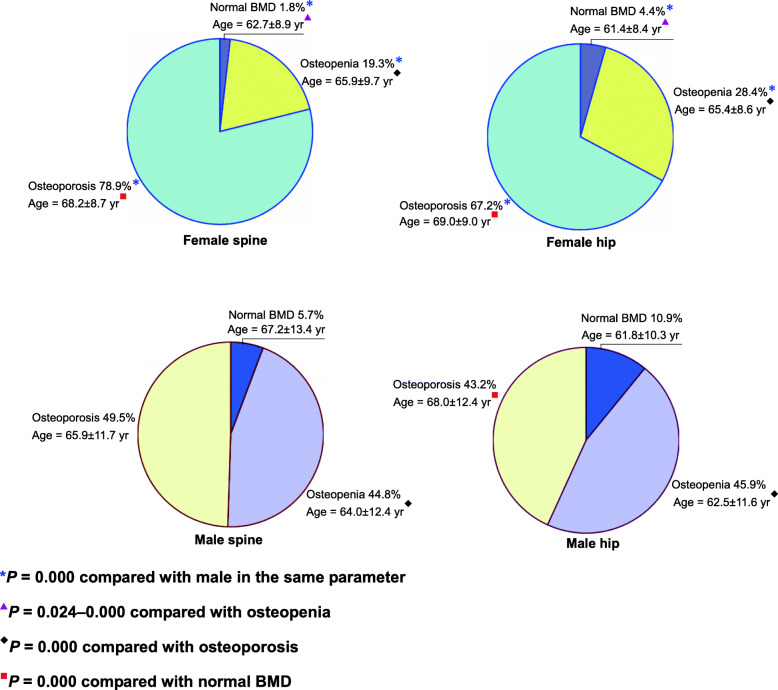
Rates of osteoporosis, osteopenia and normal BMD in fractures using gender specific *T-scores* and mean age ± SD

Among women with various types of fractures, the prevalence of osteoporosis in lumbar spine and hip was 78.9 and 67.2 % (Fig. 2), respectively, the prevalence of osteopenia was 19.3 and 28.4 % respectively and the percentage of normal BMD was 1.8 and 4.4 % respectively. There are significant differences in the mean age ± SD between osteoporosis, osteopenia, and normal BMD for both sites in women. Most female fracture patients suffer from osteoporosis and are older. Among men with various types of fractures, the prevalence of osteoporosis in lumbar spine and hip was 49.5 and 43.2 % respectively, the prevalence of osteopenia was 44.8 and 45.9 % respectively and the percentage of normal BMD was 5.7 and 10.9 % respectively. Rates of osteoporosis, osteopenia, and normal BMD for a given skeletal site significantly differed between men and women in these fracture groups.

### Fracture HR

Table 2 shows the fracture HR for female and male subjects, as determined using multivariate Cox regression analysis. BMDs and *T*-scores were stratified in descending order and analyzed. The results showed that in both male and female participants, both univariate analysis (unadjusted model) and covariance analysis (adjusted model) indicated that fracture risk ratios greatly increased with decreasing BMD and *T*-score. With a decline in PA spine BMD, HRs in adjusted model were 2.73 (95 % CI: 2.50–2.99) and 3.61 (95 % CI: 2.99–4.36) for women and men, respectively. In adjusted model, 1-point decrement in *T*-score at PA spine resulted in HRs of 1.72 (95 % CI: 1.65–1.78) and 1.68 (95 % CI: 1.59–1.78) for women and men, respectively.

**Table. 2:** Multivariate Cox regression analysis of the HR for clinical patients with osteoporotic fractures

Variable	Female (2423 pair)		Male (901 pair)	
	Unadjusted model ^a^	Adjusted model ^b^	Unadjusted model ^a^	Adjusted model ^c^
	HR (95 % CI)	HR (95 % CI)	HR (95 % CI)	HR (95 % CI)
PA-BMD	3.08 (2.83–3.36)	2.73 (2.50–2.99)	4.18 (3.51–4.99)	3.61 (2.99–4.36)
Hip-BMD	3.24 (2.96–3.55)	2.83 (2.57–3.12)	4.67 (3.86–5.65)	4.12 (3.34–5.08)
PA *T*-score	3.09 (2.84–3.36)	2.73 (2.50–2.99)	4.36 (3.64–5.22)	3.76 (3.10–4.56)
Per − 1 *T*-score	1.73 (1.67–1.80)	1.72 (1.65–1.78)	1.76 (1.68–1.85)	1.68 (1.59–1.78)
Hip *T*-score	3.24 (2.96–3.55)	2.83 (2.57–3.12)	4.62 (3.83–5.59)	4.06 (3.29–5.00)
Per − 1 *T*-score	1.60 (1.55–1.66)	1.63 (1.58–1.69)	1. 67 (1.59–1.76)	1.60 (1.51–1.70)

*PA-BMD* posteroanterior (PA) spine bone mineral density, *95 % CI *95 % confidence interval

^a^Univariate analysis

^b^ Adjusted for age, age at menopause, years since menopause, height, weight and body mass index

^c^ Adjusted for age, height, weight and body mass index

## Discussion

This case–control study involved patients with clinical fragility fracture as cases and individuals without low-impact fractures as controls; cases and controls were fully matched for age and sex at a ratio of 1:1. Comparisons between case and control groups indicated that in both men and women, the mean BMDs and *T*-scores at different skeletal sites were significantly lower in case group than in control group. In vertebral fracture subgroup, 61 % of women and 55 % of men had multiple vertebral fractures, and average lumbar BMDs in both men and women were significantly lower than those in hip and other fracture subgroups. In other fracture subgroup, 85 % of women and 80 % of men had distal forearm and wrist fractures, and average age was the lowest in this subgroup. However, mean BMDs and *T*-scores at each skeletal site were highest in other fracture subgroup, and significantly differed from those in vertebral and hip fracture subgroups. The younger age and relatively greater mean BMDs and *T*-scores among patients with distal forearm fractures may be attributable to the fact that during falls, younger patients were able to extend their arms faster in order to protect other parts of the body. However, with increasing age, the speed at which arms are extended during falls decreases [31], and therefore, the incidence of distal forearm fractures may decrease, while the probability of hip fracture may increase, after age of 65 years, especially in women. Thus, fragility fractures of distal forearm are common among older people with relatively young age and a relatively healthy body [32].

In this study, we used a proprietary sex-specific BMD reference database [27, 28], with representative *T*-scores, that we had established previously by ourselves. Thus, diagnoses of osteoporosis, osteopenia, and normal BMD in our present study were accurate and reliable. We hope that through our articles not only exhibit the relationship between BMD and fragility fracture risk in native Chinese, but also show more representative research results to colleagues, which can be meaningful contribution to construct Chinese data. Our results showed that among women with clinical fragility fractures in Changsha, China, the prevalence of osteoporosis of lumbar spine and hip was 78.9 and 67.2 % respectively. However, among men with clinical fragility fractures, the prevalence of osteoporosis of lumbar spine and hip was 49.5 and 43.2 % respectively. This suggested that osteoporosis is more common in women with fragility fractures than in men. However, the prevalence of fragility fracture was higher in men with osteopenia than in women, and the distribution of BMD *T*-scores among patients with clinical fragility fractures exhibited obvious sex-based differences. A follow-up population-based study showed that osteoporosis was present in 44 % of women with non-vertebral fractures and 64 % of women with hip fractures, but in only 21 % of men with non-vertebral fractures and 39 % of men with hip fractures [33]. Our results confirmed that clinical fragility fractures were more common among patients (both women and men) with osteoporosis, but the difference in both sexes seemed to be similar. Despite an increase in the incidence of fractures in postmenopausal women with osteoporosis, most fragility fractures (60–68 %) occur in non-osteoporotic women [34], and this ratio was even higher in a study that used peripheral bone BMD *T*-scores analysis [33]. Higher incidence of fragility fractures among osteopenic individuals may be attributable to the fact that osteopenia is much more prevalent than osteoporosis [34–36]. BMD is the main important determinant of osteoporotic fractures; other factors include aging, low BMI, history of falls, previous fracture, smoking, excessive alcohol consumption, long-term glucocorticoid use, long-acting benzodiazepine or anticonvulsant therapy, hypogonadism, long-term caffeine consumption, stroke, diabetes, non-alcoholic fatty liver, low grip strength, high-fat diet, complications, and comorbidity [7, 22, 37–41]. Increased body weight, walking, physical activity, increased muscle strength, and greater intake of fruits and vegetables may reduce the risk of fracture [22, 40, 42].

As previously stated, BMD is the main important determinant of osteoporotic fractures. In general, HRs were smaller after model adjustment, indicating that the effects of BMD and *T*-scores on fracture risk decline after elimination of the effects of covariates such as age, age at menopause, years since menopause, height, weight, and body mass index. We stratified *T*-scores using 1-point decrements, and obtained HRs of 1.63–1.72 among women; this indicates that fracture risk in women increased by 1.6–1.7 times for every 1-point decrease in *T*-score. However, in men, this risk increased by 1.6–2.0 times. Our results are similar to the results of many epidemiological follow-up studies [23–26, 33].

Since this study was not a follow-up investigation, its primary limitation is that the results obtained may not necessarily represent a causal relationship. Another limitation of this study is that histories of other related diseases and drug use, family history of fracture, and bone turnover markers related to bone metabolism were not assessed. All these factors are also associated with fragility fracture risk [22, 38, 43].

## Conclusions

Based on the relationship investigation between BMD measurement parameters and risk of fragility fractures in mainland China, our findings state that risk of clinical osteoporotic fracture increases with decreasing BMD measurement parameters and anthropometric indicators, and fracture HR exhibits obvious sex- and site-based differences. These could provide helpful guidance for clinicians to prevent fragility fractures.

## Data Availability

All data analyzed during this study are included in this published article.

## Abbreviations

BMD: Bone mineral density
DXA: Dual-energy X-ray absorptiometry
HR: Hazard ratio
PA: Posteroanterior
RMSCV: Root-mean-square coefficient of variation
WHO: World Health Organization
CI: Confidence interval
PA-BMD: Posteroanterior (PA) spine bone mineral density.
